# The complete chloroplast genome of *Ocimum gratissimum* from India – a medicinal plant in the Lamiaceae

**DOI:** 10.1080/23802359.2021.1889413

**Published:** 2021-03-17

**Authors:** Raju Balaji, Kumar Ravichandiran, Tanuja, Madasamy Parani

**Affiliations:** Center for DNA Barcoding, Department of Genetic Engineering, SRM Institute of Science and Technology, Kattankulathur, India

**Keywords:** Complete chloroplast genome, Lamiaceae, *Ocimum gratissimum*, Tulsi

## Abstract

*Ocimum gratissimum* L. is an important medicinal species with several therapeutic applications. It is used in traditional medicine as a single drug and in formulations. We generated the complete chloroplast genome sequence of *O. gratissimum* by using Illumina paired-end sequencing data. The *O. gratissimum* chloroplast genome is 152,469 bp in length, containing a large single copy (LSC) region of 83,614 bp and a small single copy region (SSC) of 17,607 bp, separated by a pair of inverted repeats (IRs) of 25,624 bp. The genome contains 138 unique genes, including 85 protein-coding, 45 tRNA, and eight rRNA genes. Among them, six genes have one intron each, and two genes contain two introns. The overall GC content of the chloroplast genome is 37.8%, while the corresponding values of LSC, SSC, and IR regions are 35.6%, 31.7%, and 43.2%, respectively. Phylogenetic analysis with the complete chloroplast genomes of other related species revealed that *O. gratissimum* is fully resolved in a clade with other *Ocimum* species classified to the family Lamiaceae.

The genus *Ocimum* includes more than 150 species, and most of them are rich in therapeutic value (Willis 1966). The leaves of *Ocimum* species are known to have antimicrobial and anti-inflammatory properties, and to serve as a remedy for headache, fever, common cold, skin aliments, diabetes, vomiting, nausea, etc. Some species are also consumed as health food supplements due to their antioxidant and anti-allergic properties (Chopra et al. 1956; Prakash and Gupta 2005). *Ocimum gratissimum* L. is an important medicinal plant used to treat microbial infections, cephalalgia, nervous disorder, neuralgia, and rheumatism (Mohanraj et al. 2018). In this study, we sequenced and assembled the complete chloroplast genome of *O. gratissimum* using Illumina pair-end sequencing data to contribute to the systematics and evolutionary history of this taxon.

Fresh leaves of *O. gratissimum* were collected from the Foundation for Revitalization of Local Health Traditions (FRLHT), Bengaluru, India (GPS coordinates: 13°07′24.5″N, 77°32′52.3″E). The herbarium voucher specimen (SRMH000142) was deposited in the SRM IST Herbarium at the SRM Institute of Science and Technology, Kattankulathur, India (Herbarium curator: Dr. E. Pandian, Email: pandiane@srmist.edu.in). Total genomic DNA from *O. gratissimum* was extracted using the CTAB method (Doyle and Doyle 1987), with some modifications (Poovitha et al. 2016). A whole-genome DNA sequencing library was constructed using the Nextera XT Library Prep Kit. The library was sequenced on the Illumina NextSeq 500 platform (Illumina Inc., San Diego, CA), and 3.63 Gb of paired-end sequencing data was obtained. The chloroplast genome of *O. gratissimum* was assembled using NovoPlasty (k-mer 31) with *O. tenuiflorum* L. (NC_043873.1) as a reference seed sequence (Dierckxsens et al. 2017). The assembled *O. gratissimum* chloroplast genome was annotated with DOGMA (Wyman et al. 2004) and GeSeq (Tillich et al. 2017) using the chloroplast genomes of *O. tenuiflorum* (NC_043873.1) and *O. basilicum* L. (NC_035143.1) as reference sequences. The predicted transfer RNAs (tRNAs) were confirmed by tRNAscan-SE 2.0 (Lowe and Chan 2016). The complete chloroplast genome of *O. gratissimum* with annotations was submitted to GenBank (Accession No. MW348919), and the raw reads were deposited in the GenBank Sequence Read Archive (Accession No. SRR13206921).

The complete chloroplast genome of *O. gratissimum* is 152,469 bp with a mean coverage of 888x. It has a typical quadripartite structure, including a large single copy (LSC) region of 83,614 bp, a small single copy (SSC) region of 17,607 bp, and a pair of inverted repeats (IRs) of 25,624 bp. The chloroplast genome contains 85 protein-coding genes, 45 tRNA genes, and eight rRNA genes. Among them, six genes (*atpF, rpoC1, ndhK, rpl2, ndhA,* and *ndhB*) are single-intron genes, and two genes (*ycf3* and *clpP*) contain two introns. The overall GC content is 37.8%, while LSC, SSC, and IR regions are 35.6%, 31.7%, and 43.2%, respectively.

A neighbor-joining tree with 1000 bootstrap replicates was performed using MEGA v7.0 (Kumar et al. 2016) from the alignments created by the MAFFT program (Katoh and Standley 2013). *Nicotiana tabacum* L. (Solanaceae) and *Arabidopsis thaliana* L. (Brassicaceae) were designated as outgroups, and 18 published chloroplast genomes from the Lamiaceae were included as ingroup taxa. The phylogenetic analysis fully resolved *O. gratissimum* in a clade with the closely related *O. basilicum,* in a clade also containing *O. tenuiflorum* (Figure 1). *Ocimum* was sister to the genus *Perilla* (Figure 1). This complete chloroplast genome of *O. gratissimum* can be subsequently used for phylogenetic analysis, DNA barcoding, and chloroplast genetic engineering studies of genus *Ocimum* and Lamiaceae family.

**Figure 1.**
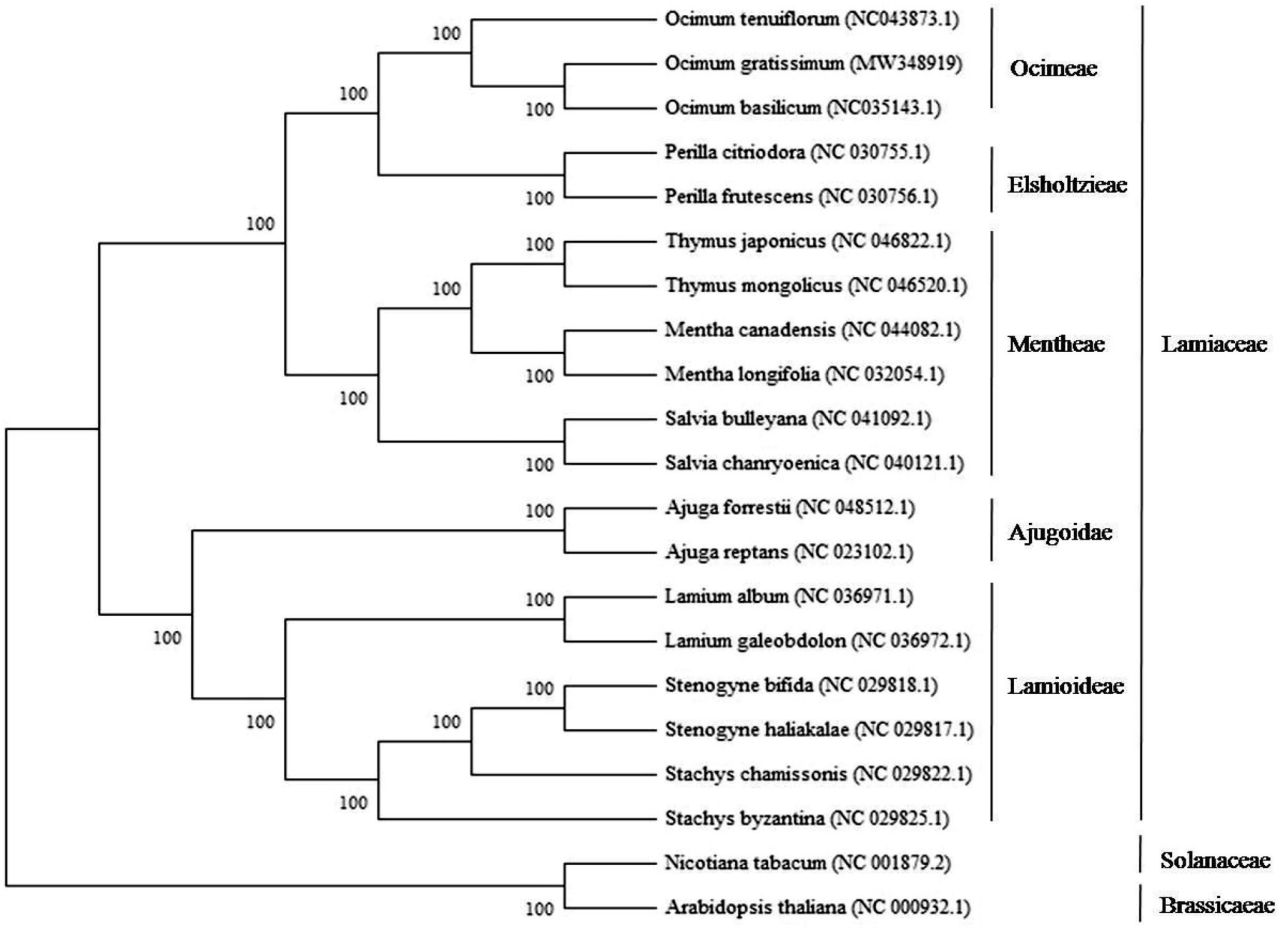
Neighbor-joining (NJ) tree based on the whole chloroplast genome sequences of 21 species including *Nicotiana tabacum* L. and *Arabidopsis thaliana* L. as outgroups. The complete chloroplast genome sequences were aligned using MAFFT online version (https://mafft.cbrc.jp/alignment/server/) and subjected to generating NJ phylogenetic tree by MEGA v7.0 (Kumar et al. 2016). The bootstrap support values (>50%) from 1000 replicates are indicated in the nodes.

## Data Availability

The data that support the findings of this study are openly available in GenBank at https://www.ncbi.nlm.nih.gov/genome under the accession numbers [NC_048512, NC_023102, NC_036971, NC_036972, NC_029818, NC_029817, NC_029825, NC_029822, NC_030755, NC_030756, NC_035143, NC_043873, NC_046822, NC_046520, NC_044082, NC_027784, NC_041092, NC_040121, and NC_001879]. The complete chloroplast genome assembled in this study was deposited in GenBank under the accession number MW348919. The NGS sequencing data files are available from the BioProject, SRA, and Bio-Sample ID under the accession numbers PRJNA682904, SRR13206921, and SAMN17013753, respectively.
